# Supplementary material for: Slc7a8 Deletion Is Protective against Diet-Induced Obesity and Attenuates Lipid Accumulation in Multiple Organs
Source: Biology (Basel). 2022 Feb 16;11(2):311. doi: 10.3390/biology11020311 (PMC8869389; doi:10.3390/biology11020311)
Supplement: Supplementary file 1 [file biology-11-00311-s001.zip › biology-1507682-supplementary.pdf]

Supplementary materials

Supplementary methods

S1: Genotyping of mice

Genomic DNA was extracted from tail biopsy of mouse pups using the KAPA Mouse Genotyping Kit (*Wilmington, Massachusetts, United States of America*) and the KAPA Express Extract Protocol. The extractions were performed in a volume of 100 µl and was set up as follows: 88 µl PCR-grade water, 10 µl of 10X KAPA Extract Express buffer, 2 µl of 1 U/µl KAPA Express Extract enzyme and approximately 2 mm of mouse tail tissue. Enzymatic lysis was performed in the Applied Biosystems 9700 thermal cycler (*Foster City, California, United States of America*) at 75 °C for 10 minutes and enzyme inactivation at 95 °C for 5 minutes. The DNA extracts were subsequently diluted 10-fold in 10 mM TRIS-HCL (pH 8.5).

To determine the wildtype, heterozygous and knockout *SLC7A8* genotypes, the following gene-specific primer sequences were used: 5′-CAAATGCCAGCTGTCCTGACCTCAC-3′ forward primer for the wildtype allele, 5′-GGGTGGGATTAGATAAATGCCTGCTCT-3′ forward primer for the knockout allele and 5′-CAGACTTAGGGATGGTGACGCCTAG-3′ for the common reverse primer. All oligonucleotides used in the study were synthesised by Integrated DNA Technologies (*Coralville, Iowa, United States of America*). The PCR reaction mixture consisted of 6.5 µl of PCR-grade water, 12.5 µl of the KAPA2G Fast Genotyping buffer, 1.25 µl of both the 10 µM wildtype forward primer and 10 µM knockout forward primer, 2.5 µl of 10 µM common reverse primer and 1 µl of the diluted DNA extract. The PCR amplifications were performed in a total volume of 25 µl and cycled in the ABI Applied Biosystems 9700 thermal cycler. The thermal cycling conditions used were as such: 95 °C for 3 minutes followed by 95 °C for 15 seconds, 60 °C for 15 seconds, 72 °C for 15 seconds and a final extension for 2 minutes at 72 °C. After amplification, 10 µl of each amplicon was separated on a 2% agarose gel alongside a Thermo Scientific FastRuler Low Range DNA ladder (*Waltham, Massachusetts, United States of America*). Electrophoresis was performed in 1 x TAE (diluted from UltraPure 10 x TAE buffer (*ThermoFischer Scientific, Waltham, Massachusetts, United States of America*) at 120V for 40 minutes. The gel was stained with Ethidium Bromide Solution, Molecular Grade (*Promega, Madison, Wisconsin, United States of America*) and viewed under UV light using the Molecular Imager Gel Doc XR System (*Bio-Rad, Hercules, California, United States of America*). The expected amplicon sizes were 206bp for the wildtype allele and 390bp for the knockout allele. Only wildtype and knockout mice for the *SLC7A8* gene were used in the study.

Supplementary figures

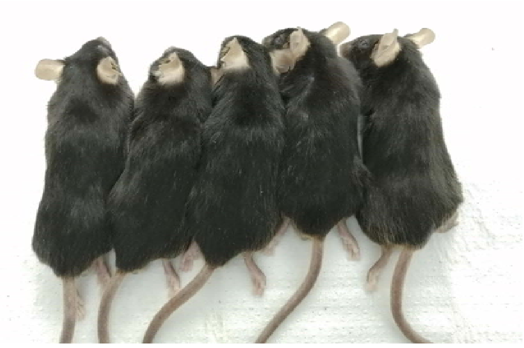

(**a**)

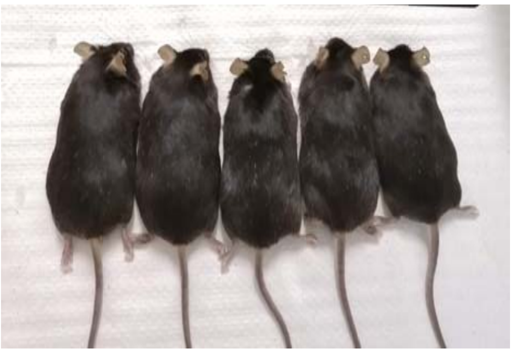

(**b**)

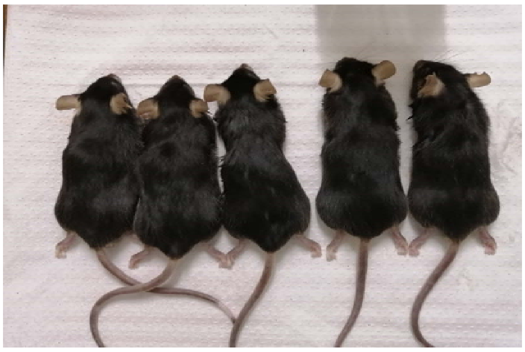

(**c**)

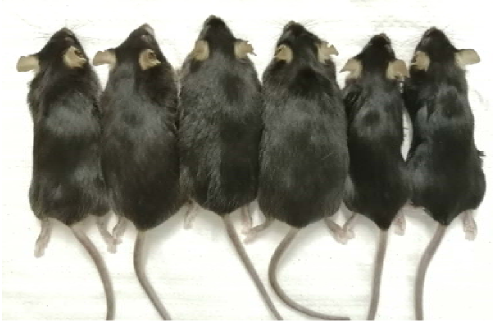

(**d**)

Figure S1: Mice used in the study. (**a**) Represents KOCD14 mice. (**b**) KOHFD14 mice (**c**) WTCD14 mice (**d**) WTHFD14 mice. The images demonstrate that KOHFD14 and WTHFD14 mice are larger in size than their control diet counterparts, KOCD14 and WTCD14, respectively.

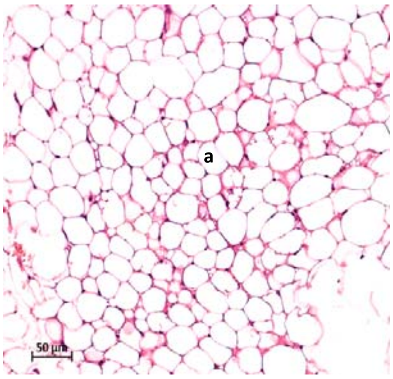

(**a**)

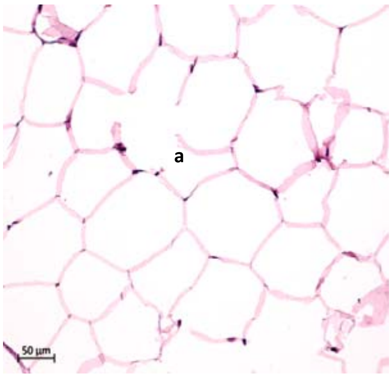

(**b**)

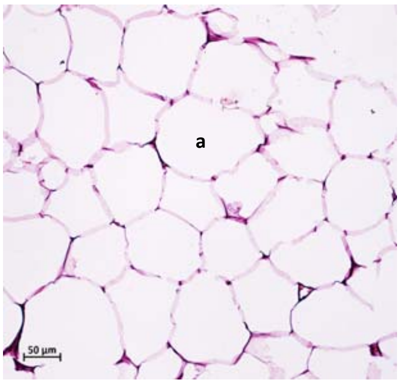

(**c**)

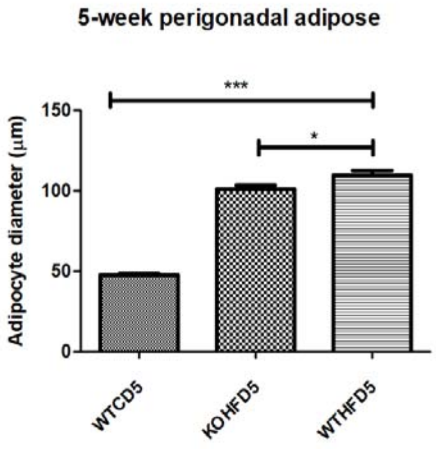
5-week perigonadal adipose
***
*
Adipocyte diameter (μm)
150
100
50
0
WTCD5
KOHFD5
WTHFD5

(**d**)

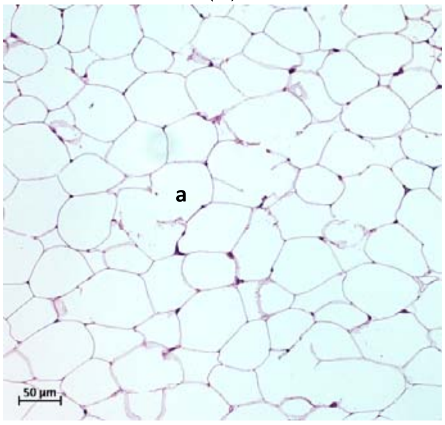
a
50 μm

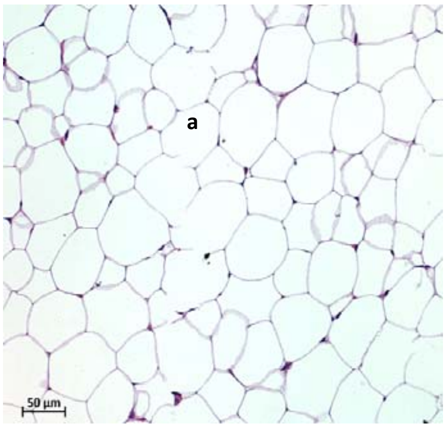
a
50 μm

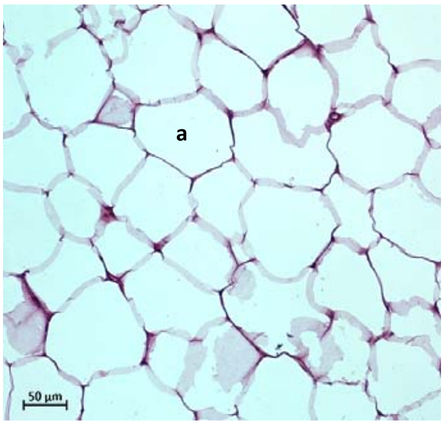
a
50 μm

(**e**) (**f**) (**g**)

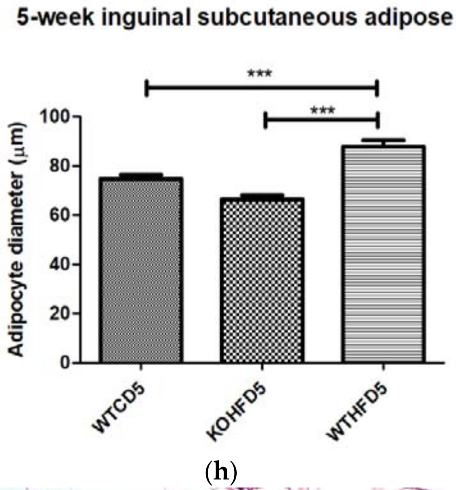
5-week inguinal subcutaneous adipose
***
***
Adipocyte diameter (μm)
100
80
60
40
20
0
WTCD5
KOHFD5
WTHFD5

(**h**)

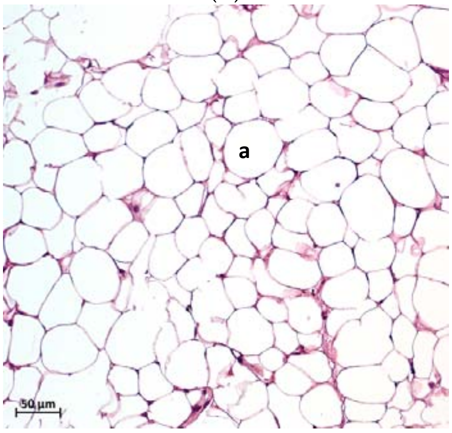
a
50 μm

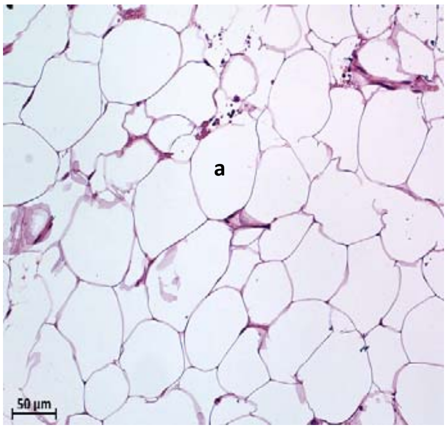
a
50 μm

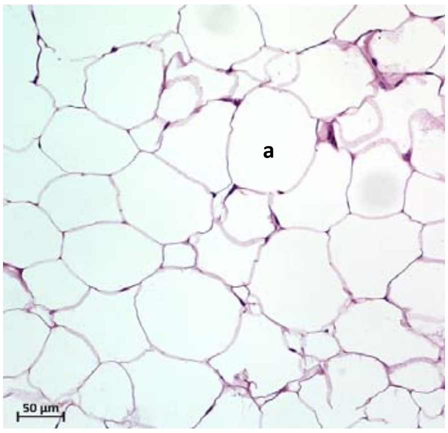
a
50 μm

(**i**) (**j**) (**k**)

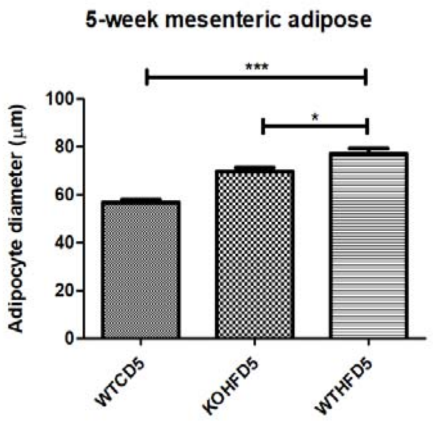

(**l**)

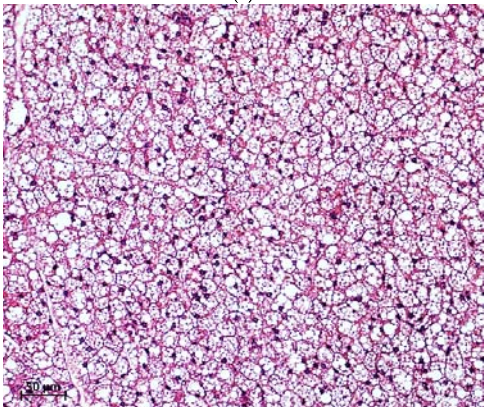

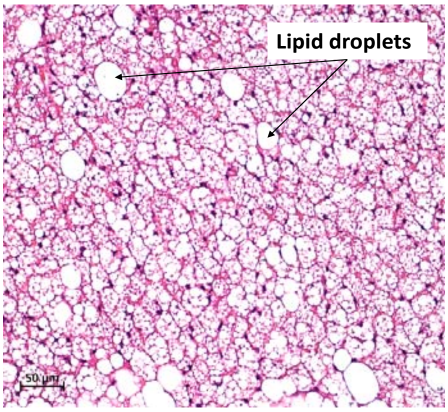

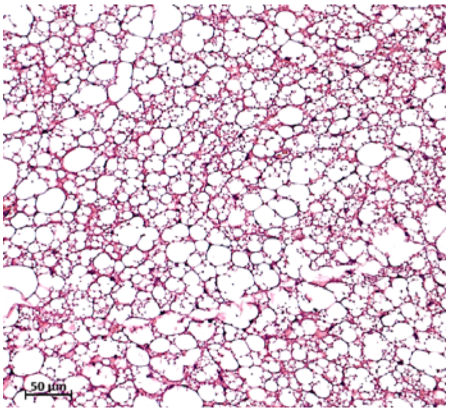

(**m**) (**n**) (**o**)

Figure S2: Adipocyte hypertrophy at 5 weeks. Adipocyte diameter of WTHFD (**c**) in pWAT was significantly larger than WTCD (**a**) ($p<0.001$) and KOHFD (**b**) ($p<0.05$). In iWAT, WTHFD (**g**) adipocyte hypertrophy was significantly greater ($p<0.05$) than WTCD (**e**) and KOHFD (**f**). WTHFD (**k**) in mWAT showed significantly larger adipocytes than WTCD (**i**) ($p<0.001$) and KOHFD (**j**) ($p<0.05$). Accumulation of enlarged lipid droplets were observed in WTHFD (**o**) than WTCD (**m**) and KOHFD (**n**).

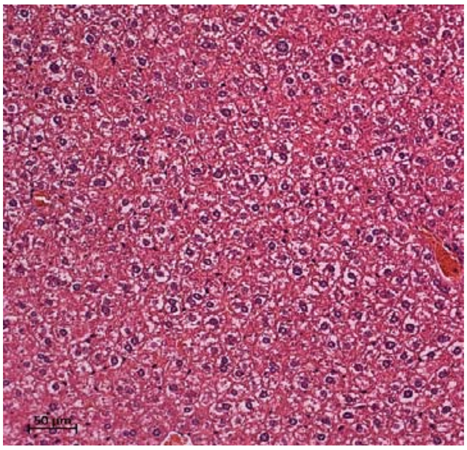

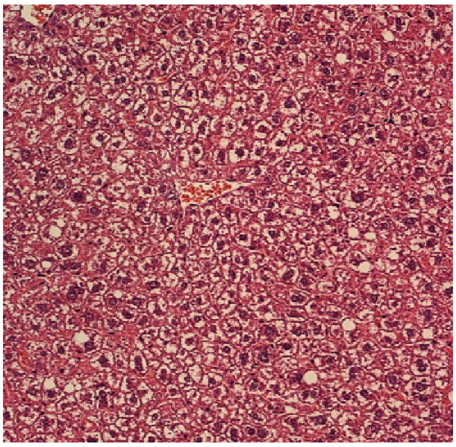
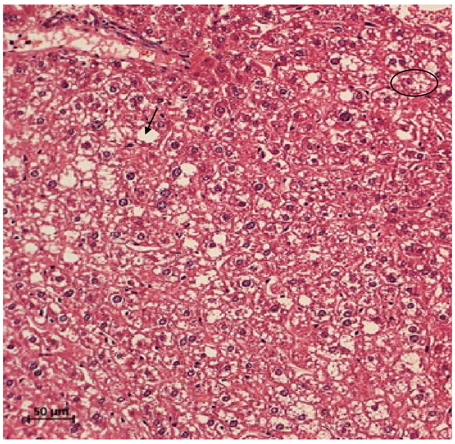

(**a**) (**b**) (**c**)

Figure S3: Lipid droplets in the liver at 5 weeks. WTHFD (**c**) and KOHFD (**b**) had lipid droplets in the tissue, while none were observed in WTCD (**a**).

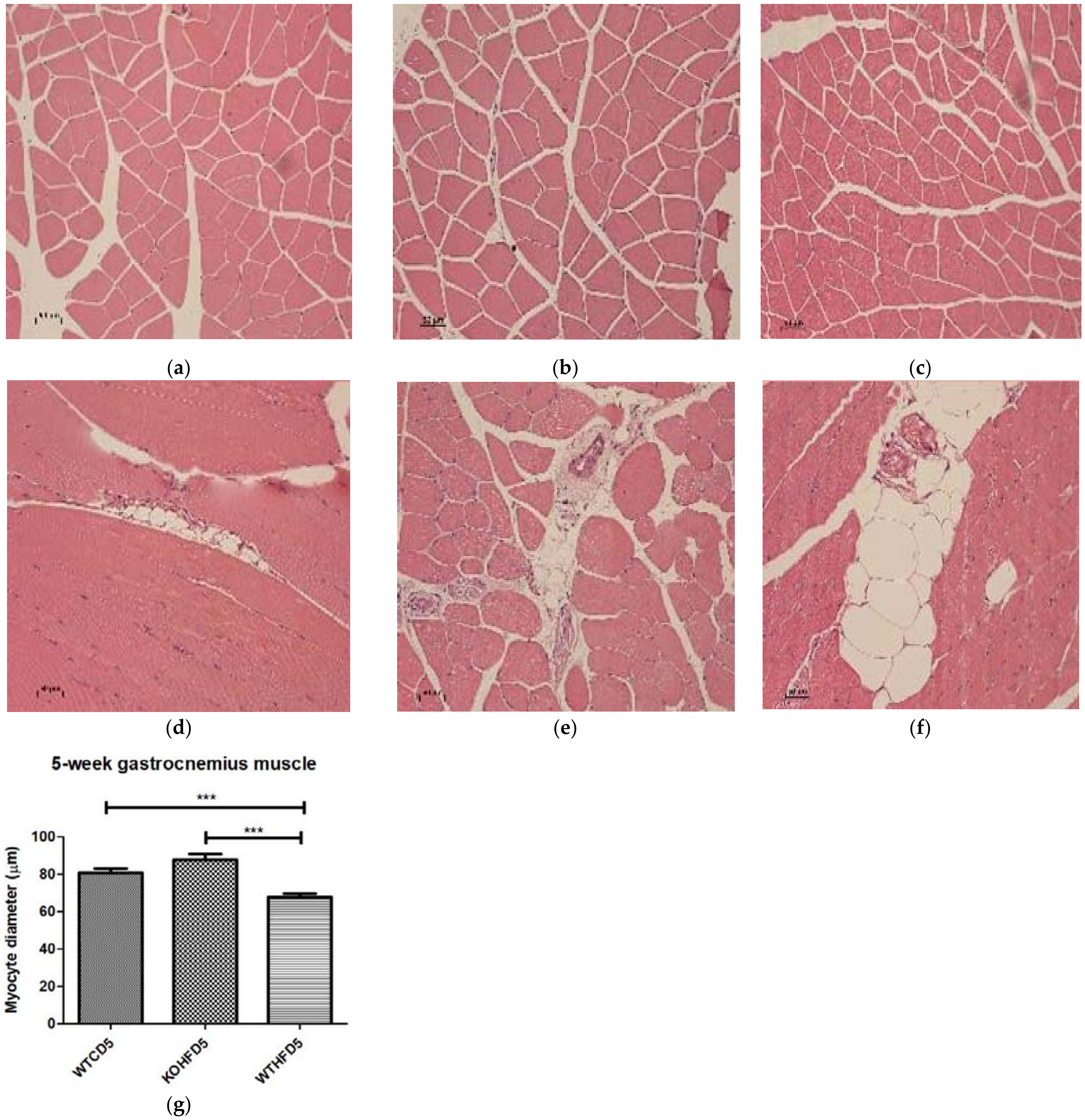

Figure S4: Significantly larger myocytes (p<0.001) (**g**), were observed in the WTCD (**a**) and KOHFD (**b**) in comparison to those in the WTHFD (**c**). The distribution of peri-muscular adipose tissue shows that greater accumulation of the adipose was observed in WTHFD (**f**) compared to WTCD (**d**) and KOHFD (**e**).

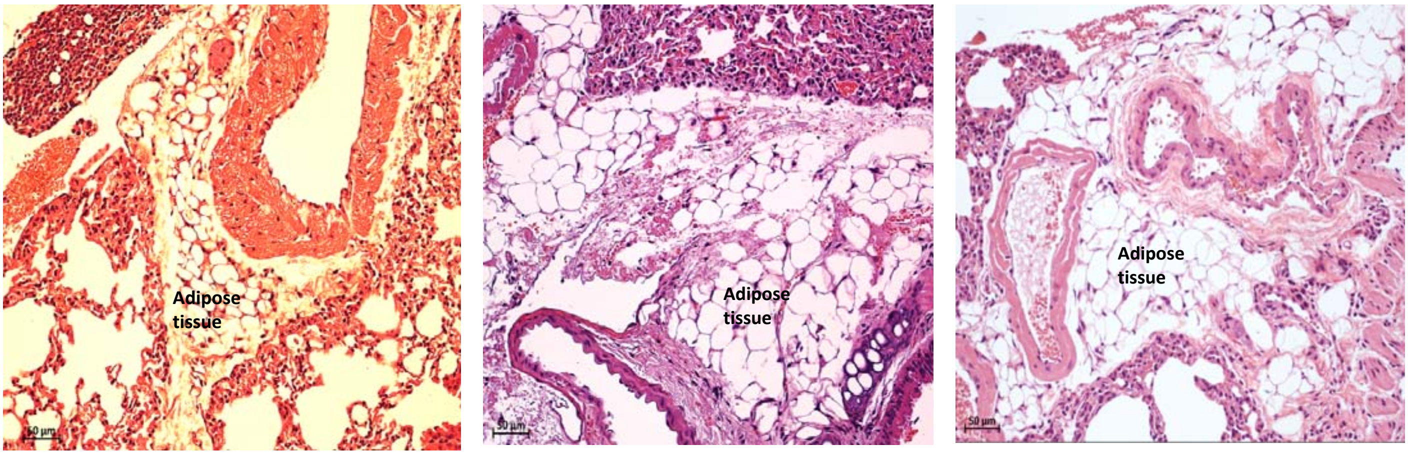

(**a**) (**b**) (**c**)

Figure S5: Accumulation of adipose tissue in the lungs. Greater accumulation was observed in WTHFD (**c**) and KOHFD (**b**) in comparison to WTCD (**a**).

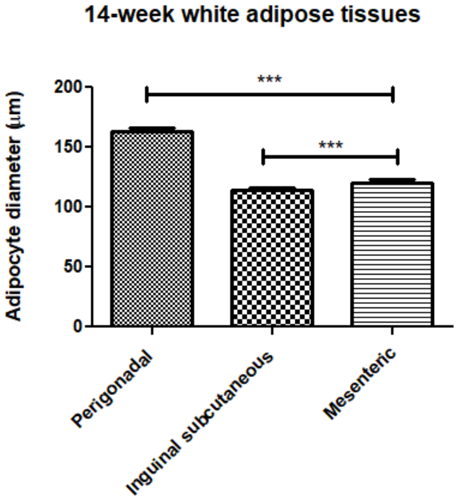

Figure S6: Perigonadal adipose tissue in WTHFD is significantly larger (p<0.001) than inguinal and mesenteric adipose tissues.
